# *In vitro* and *in vivo* antidiabetic activity of bitter honey in streptozotocin-nicotinamide-induced diabetic Wistar rats

**DOI:** 10.25122/jml-2022-0099

**Published:** 2023-01

**Authors:** Joshna Koodathil, Gopal Venkatachalam, Kavitha Bhaskaran

**Affiliations:** 1 Department of Pharmacognosy, College of Pharmacy, Mother Theresa Post Graduate and Research Institute of Health Sciences, Puducherry, India

**Keywords:** bitter honey, *in vitro*, *in vivo*, antidiabetic

## Abstract

Diabetes mellitus is a metabolic syndrome considered one of the life-threatening diseases in the last two decades. This research aimed to investigate the anti-diabetic potential of bitter honey collected from Nilgiris using both *in vitro* and *in vivo* methods. The mineral content of bitter honey was also estimated using atomic absorption spectrophotometer. Bitter honey had a higher amount of zinc and copper, while heavy metals like lead, nickel, and cadmium were below the detection limit. The *in vitro* antidiabetic study was performed using *alpha-amylase* and *alpha-glucosidase* inhibition methods. Acute toxicity (OECD 423) was conducted in female Wistar rats to determine the lethal dose of bitter honey. The antidiabetic activity was carried out in type-2 diabetic Wistar Albino rats induced with streptozotocin and nicotinamide. The experimental rats were categorized into five groups (n=8): the normal group, the diabetic control group, standard glibenclamide-treated diabetic group, bitter honey 200 mg/kg, and 400 mg/kg b.w. treated diabetic group. After the treatment period (28 days), blood samples were collected for biochemical studies, and the pancreas was dissected for histopathological studies. The *in vitro* antidiabetic studies revealed the antidiabetic potential of bitter honey compared to standard acarbose. Treatment of diabetic rats with bitter honey revealed a statistically significant reduction (P<0.05) in the levels of fasting blood glucose (FBG) compared to untreated diabetic rats. This was accompanied by an elevated HDL and a decrease in LDL, VLDL, triglycerides, total cholesterol, SGOT, SGPT, urea, and creatinine. Histopathological changes in the pancreas indicated a marked improvement in a dose-dependent manner. The study concluded that bitter honey could potentially decrease the levels of FBG in diabetic rats and the various biochemical and histopathological abnormalities associated with diabetes mellitus.

## INTRODUCTION

Diabetes mellitus develops because of deficiency (or) impairment in insulin secretion. India has the world's second-largest population with diabetes. Type-II diabetes (or) non-insulin dependent diabetes mellitus (NIDDM) is the most prevalent type of diabetes. The hydrolysis of complex carbohydrates is facilitated by *alpha-amylase* and *alpha-glucosidase*, followed by intestinal glucose uptake, resulting in increased blood glucose levels. Inhibition of the two enzymes helps decrease blood glucose levels after a carbohydrate diet and plays an essential role in managing NIDDM [1–4]. Complications affecting the heart, eyes, kidneys, and nerves are also associated with diabetes mellitus [5]. Long-term use of the antidiabetic drugs currently available on the market has undesired side effects [6]. Contrary to this, natural products have high efficacy and low incidence of side effects. Indigenous medicine has gained more interest worldwide due to its importance in preventing and managing various diseases [7, 8].

Honey is rich in phenolic compounds attributed to its medicinal properties [9]. Recently, honey research has gained much interest due to its cardioprotective, hepatoprotective, hypoglycaemic, antioxidant, and antihypertensive effects [10]. Furthermore, in a study conducted on normal individuals and patients with hyperlipidemia, natural honey consumption decreased cholesterol, CRP (C-reactive protein), and homocysteine levels. Honey has been recognized and appreciated as both food and medicine from antiquity. The usage of honey is even advocated in the Quran and Bible [11, 12]. The antidiabetic effects of different varieties of sweet honey have been reported. In pancreatic hamster cells, Gelam honey extracts reduced oxidative stress-induced inflammation [13]. Nigerian honey was shown to lower lipid levels and blood glucose levels in diabetic rats induced by alloxan [14]. Diabetic rats fed Tulang honey and glibenclamide, or metformin had improved blood glucose control [15].

Honey contains various elements like calcium, sodium, magnesium, potassium, chlorine, iron, zinc, and copper beneficial to human health. The mineral content of honey is an indicator of the geographical source of honey. It is also helpful in determining the presence of heavy metals, like lead, cadmium, and nickel, that are hazardous to health [16]. Researchers have also reported the beneficial effect of zinc and honey for treating diabetes [17, 18].

Apis dorsata bees build hives on the *Syzygium cumini* tree and produce bitter honey. This type of honey has a characteristic bitter aftertaste, is dark brown in color, and is mainly found in the Nilgiri region of Tamil Nadu, India, harvested by local Alu Kurumba tribes. The most predominant pollen species found in this variety of honey is *Syzygium* pollen which belongs to the Myrtaceae family. *Syzygium* species exhibit antioxidant, anti-diabetic, and antilipidemic activities. The antidiabetic effect of *Syzygium cumini* has also been mentioned in Ayurvedic Pharmacopoeia and has been practiced over 130 years ago [19]. *Syzygium cumini* was introduced into Western medicine in the mid-nineteenth century due to its anti-diabetic effect. An ethnomedical survey conducted among local Alu Kurumba tribes of Nilgiri recorded that bitter honey is used to treat cough, stomach ache, wounds, and conditions resembling diabetes.

The extensive literature review revealed that bitter honey produced in the Nilgiri biosphere is scientifically underexploited. Therefore, the present research focused on evaluating the *in vivo* and *in vitro* antidiabetic potential of bitter honey.

## MATERIAL AND METHODS

### Collection of bitter honey

The sample was collected from the honey hunter Alu Kurumba tribes of Nilgiris in 2018. The honey samples were stored at 4ºC until further analysis.

### Elemental analysis of bitter honey

The elemental analysis of bitter honey was carried out using atomic absorption spectrophotometer (AAS Avante-PM). The content of copper, iron, calcium, zinc, lead, cadmium, and nickel was determined by comparing it with the standard solutions of each element (Merck). Air acetylene was used as a carrier gas at 2300℃ [20].

### *In vitro* antidiabetic assay

*Alpha-glucosidase* was procured from HIMEDIA Laboratory, Mumbai, India. Pancreatic *alpha-amylase* was procured from Sigma Aldrich, USA.

### *In vitro* assay of α-amylase inhibition

The *alpha-amylase* inhibition of bitter honey (0.2–10 mg/mL) was carried out using the 3,5-dinitro salicylic acid method as described by Miller, 1959 [21]. Acarbose was used as a positive control in the assay, with concentrations ranging from 0.02–1 mg/mL. The α-amylase inhibition percentage was determined, and IC50 values were calculated.

### *In vitro* assay of α-glucosidase inhibition

*In vitro* α-glucosidase inhibition of bitter honey (0.1–1 mg/mL) and standard acarbose (0.01–0.1 mg/mL) was carried out by GOD-POD method [22]. The α-glucosidase inhibition percentage was determined, and IC50 values were calculated.

The inhibition percentage of both enzymes was calculated utilizing the below equation:


$$Percentage\;Inhibition=\left[\left(Abs\;of\;C-Abs\;of\;S/Std\right)/Abs\;of\;C\right)]\times 100$$

Abs of C – absorbance of Control; Abs of S – absorbance of Sample; Abs of Std – absorbance of Standard.

The concentration of bitter honey in *in vitro* assays was selected based on similar *in vitro* studies on bee honey [23].

### *In vivo* antidiabetic study

Adult Albino male Wistar rats (180–240 g, n=40) and female Wistar rats (n=6) were purchased from Biogen Laboratory Animal Facility, Bangalore. The experimental rats were provided with a standard pellet diet procured from VRK'S Scientist Solutions, Maharashtra, and distilled water *ad libitum*.

### Acute toxicity studies of bitter honey

OECD 423 guidelines were followed in carrying out acute toxicity studies [24]. The animals were categorized into two groups, with three animals in each group. The first group of animals was given distilled water, while the second group was administered a single 2000 mg/kg b.w. bitter honey oral dose, (p.o.). The rats were then monitored for 30 minutes, 4 hours, and 24 hours for 14 days for any toxic signs, including tremors, convulsions, lethargy, diarrhea, sleep, and coma.

### Antidiabetic study

#### Induction of diabetes

The experimental animals were randomly distributed into five groups. The control rats received distilled water, and all four groups were administered Streptozotocin (STZ), 60 mg/Kg, i.p (Sisco research laboratories Pvt. Limited, India) in citrate buffer (0.1M, P^H^ 4.5). Prior to the administration of STZ, Nicotinamide (NAD), 120 mg/Kg (Sisco research laboratories Pvt. Limited, India), was prepared in normal saline and was administered intraperitoneally.

In order to withstand the initial hypoglycemic phase after STZ administration, drinking water was replaced with glucose (5%) for 24 hours while the standard pellet diet was provided throughout the study [25].

#### Determination of fasting blood glucose (FBG) levels

The FBG measurements were made using a glucometer (one-touch Verio flex). Rats with FBG levels above 200 mg/dl with signs of polyuria and polydipsia were confirmed to be diabetic and were involved in the study. The following treatment was given in oral dosing using a gavage tube to all groups for 28 days. Rats in Groups I and diabetic rats in Group II were administered orally with distilled water. Group III diabetic rats were treated with Glibenclamide standard, 0.6 mg/kg b.w., and bitter honey was administered to diabetic rats in Groups IV and V, 200 mg/kg b.w. and 400 mg/kg b.w., respectively. The Glibenclamide standard solution was prepared in 1% Carboxymethyll cellulose, while bitter honey samples were prepared by diluting in distilled water. After the experimental period (24 hours after the last dose), the animals were euthanized using an excess dose of anesthesia (thiopentone sodium, 75 mg/kg ip) [26].

#### Biochemical analysis

Measurement of Water intake and urine volume: The rats were kept in metabolic cages, and water intake and urine volume were recorded on days 0 and 28 [27].

Measurement of Fasting Blood Glucose (FBG) levels: Retro-orbital blood sampling was performed on days 0, 7, 14, 21, and 28 in collecting tubes and was centrifuged at 3000 rpm for about 10 minutes. Finally, the supernatant serum was separated and analyzed for the following biochemical parameters [28]. The measurement of FBG levels was made using a glucometer.

#### Determination of lipid profile levels

The levels of total cholesterol (TC), triglycerides (TG), and high-density lipoprotein (HDL) were carried out in semi autoanalyzer (Photometer 5010, Germany) using Agappe Kits. Friedwalds formula was used to calculate the levels of low-density lipoprotein (LDL) and very low-density lipoprotein (VLDL) as follows:


$$LDL=TC-\left[HDL-\left(TG\right)/5\right)];\;VLDL=TG/5$$

#### Estimation of liver and kidney markers

The separated serum was analyzed for the measurement of kidney and liver markers, including serum glutamic oxaloacetic transaminase (SGOT), serum glutamic pyruvic transaminase (SGPT), urea, and creatinine, using a commercially available kit (Span diagnostics limited, India).

#### Histopathological study

After collecting blood samples, the pancreas was dissected, removed, and fixed in a 10% formalin solution. Thin paraffin-embedded microtome sections of pancreatic tissues were made, stained with eosin and hematoxylin, and were observed at 400X magnification under a light microscope (Magnus MLX Plus) [29].

### Statistical analysis

In the *in vitro* study, the IC50 values were indicated as the mean of percentage inhibition±standard deviation (n=3) for standard acarbose and bitter honey. The one-way analysis of variance (ANOVA) was used to assess the *in vivo* data, followed by Tukey's multiple comparisons test to compare the results. The software used was GraphPad Prism software 9.3.0.

## RESULTS

The elemental analysis results of bitter honey are displayed in Table 1. The bitter honey sample was a good source of copper and zinc, while heavy metals like lead, cadmium, and nickel were not reported.

**Table 1:** Mineral content of bitter honey.

Sl. No	Name of the metal	Value in mg/kg
**1**	Zinc	1.65±0.03
**2**	Copper	0.82±0.02
**3**	Lead	BDL
**4**	Nickel	BDL
**5**	Cadmium	BDL

The results are Mean±Standard deviation; BDL – Below Detection Limit.

**Figure 1:**
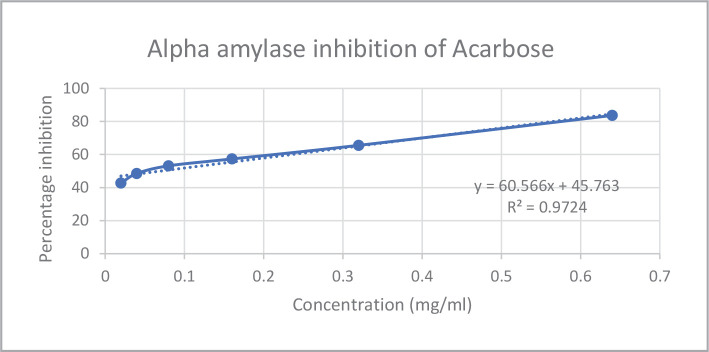
Alpha amylase inhibition of acarbose.

**Figure 2:**
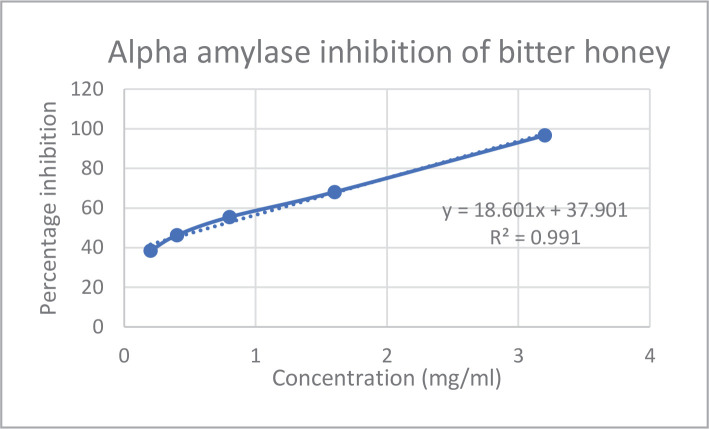
Alpha amylase inhibition of bitter honey.

**Figure 3:**
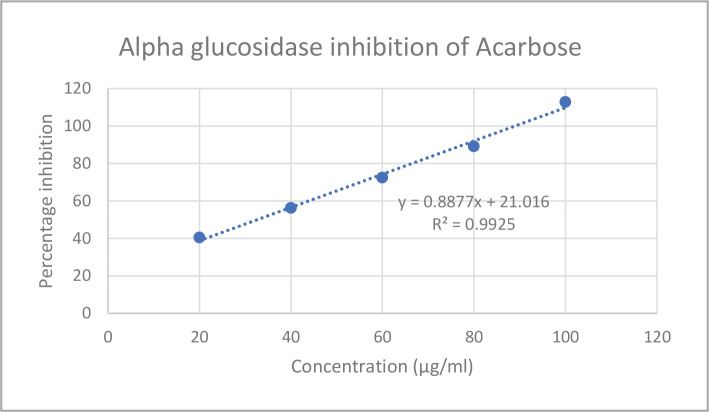
*Alpha-glucosidase* inhibition of acarbose.

**Figure 4:**
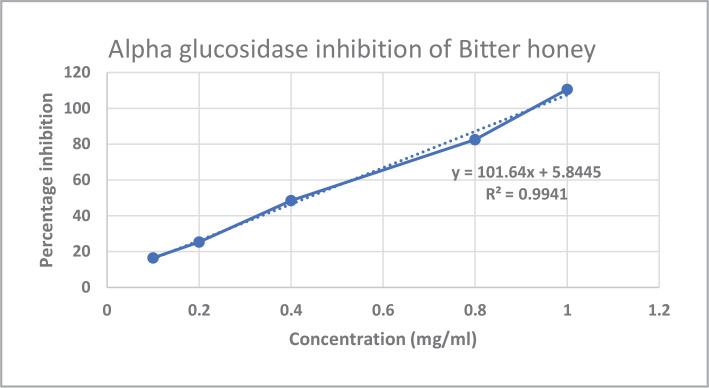
*Alpha-glucosidase* inhibition of bitter honey.

*In vitro* assays like *alpha-amylase* and *alpha-glucosidase* inhibition were used to investigate the antidiabetic potential of bitter honey. The *alpha-amylase* and *alpha-glucosidase* inhibition percentages and IC50 values of bitter honey and standard drug acarbose are represented in Figures 1–4 and Table 2, respectively. Standard drug acarbose exhibited 42.69% of *alpha-amylase* inhibition at a concentration of 0.02 mg/ml, while bitter honey at a concentration of 0.2 mg/mL exhibited an inhibition of 38.45% against the *alpha-amylase* enzyme. The *alpha-glucosidase* concentration of bitter honey at a concentration of 0.1 mg/mL showed an inhibition of 16.46%. The percentage inhibition of acarbose standard against *alpha-glucosidase* enzyme at the same concentration exhibited 112.86%.

**Table 2:** *In vitro* antidiabetic assay results of acarbose and bitter honey.

Sample	α-amylase inhibition IC50 (mg/ml)	α-glucosidase inhibition IC50 (mg/ml)
**Acarbose**	0.07±0.003	0.03±0.002
**Bitter honey**	0.72±0.110	0.44±0.006

The results are Mean±Standard deviation.

Acute oral toxicity studies were conducted on bitter honey to determine the LD50 value. A single oral administration of bitter honey (2000 mg/kg b.w.) on Wistar albino rats did not produce any behavioral changes or mortality. This indicates that the LD50 value of bitter honey would be greater than 2000 mg/kg. Bitter honey at a 2000 mg/kg dose may be classified as safe for consumption. Hence 1/5^th^ and 1/10^th^ of the maximum dose tested were selected as 400 mg/kg and 200 mg/kg for the present study. The water intake and urine output were increased in diabetic rats compared to control rats (Table 3). The water intake and urine output of diabetic rats that received the standard drug and bitter honey reduced significantly (P<0.05) compared to untreated diabetic rats.

**Table 3:** Effect of bitter honey on water intake and urine volume (mL/day).

Parameter	Group 1		Group 2		Group 3		Group 4		Group 5	
	Day 0	Day 28	Day 0	Day 28	Day 0	Day 28	Day 0	Day 28	Day 0	Day 28
**Water intake (ml/day)**	43.62±2.5	55.12±5.5	94.37±4.0	109.1±7.8	90.37±6.0	75.87±4.2*	87.62±5.4	76.75±4.8*	80.37±2.7	70.75±4.3*
**Urine volume (ml/day)**	13.12±2.7	13.37±2.1	65.12±6.9	68.12±5.9	61.37±7.4	50.15±4.9*	57.37±6.6	48.25±6.3*	55.50±3.8	45.12±5.1*

The results are Mean±S.E.M (n=8); * – Indicates a significant difference from Group II, P<0.05.

The body weight of standard drug-treated Group III diabetic rats showed improvement compared to Group II rats (Table 4). Bitter honey-treated diabetic rats did not produce any improvement (P<0.05) in body weight even after treatment for 28 days, indicating that bitter honey does not have any beneficial effect in improving the body weight of diabetic animals. Fasting blood glucose (FBG) changes observed weekly are represented in Table 5. FBG levels of diabetic Group II rats were higher compared to group I rats that received distilled water. The FBG levels were reduced significantly (P<0.05) in glibenclamide-treated rats from day 7 to 28 days, while the FBG levels of Group IV and V bitter honey-treated groups did not produce any reduction until day 14. From day 14 to day 28 of the study, the fasting blood glucose levels of the bitter honey-treated groups exhibited a significant reduction (P<0.05) compared to the FBG levels of group II untreated diabetic rats. On day 28, the FBG levels of untreated diabetic rats were elevated. When diabetic untreated rats were compared to non-diabetic rats, higher blood triglyceride levels were detected (Table 6). However, the reduction in serum triglyceride levels of standard drug and bitter honey-treated groups was similar. Diabetic rats treated with bitter honey had significantly higher levels of HDL cholesterol levels (P<0.05) compared to Group II diabetic rats (Table 7). The LDL cholesterol levels of diabetic rats treated with the standard drug and bitter honey reduced significantly (P<0.05) compared to diabetic untreated rats.

**Table 4:** Effect of bitter honey on changes in body weight.

Treatment group	Bodyweight (g)	
	On day 0	On day 28
**Group I**	231.19±5.17	257.68±5.19
**Group II**	187.18±2.52	156.71±3.50
**Group III**	209.62±4.38	220.57±4.17*
**Group IV**	189.89±1.87	177.93±1.92
**Group V**	186.79±1.88	175.82±2.27

The results are Mean±S.D (n=8); * – Indicates a significant difference from Group II, P<0.05.

**Table 5:** Effect of bitter honey on fasting blood glucose level (mmol/L).

Day	Group I	Group II	Group III	Group IV	Group V
**0**	3.91±0.09	16.56±0.19	17.01±0.16	18.55±3.63	18.12±0.19
**7**	4.01±0.07	17.42±0.17	14.59±0.21*	17.43±0.19	16.71±0.22
**14**	4.03±0.15	17.85±0.18	12.15±0.22*	15.97±0.22*	14.32±0.32*
**21**	3.99±0.13	18.42±0.72	10.36±0.67*	14.60±0.27*	12.64±0.31*
**28**	4.09±0.18	19.73±0.18	9.30±0.21*	13.33±0.25*	10.84±0.33*

The results are Mean±S.E.M (n=8); * – Indicates a significant difference from Group II, P<0.05.

**Table 6:** Effect of bitter honey on serum triglyceride levels.

Treatment group	Serum triglyceride (mmol/L)	
	Day 0	Day 28
**Group I**	0.65±0.02	0.62±0.03
**Group II**	0.94±0.04	1.09±0.06
**Group III**	0.90±0.03	0.79±0.03*
**Group IV**	1.00±0.02	0.82±0.02*
**Group V**	0.92±0.03	0.79±0.01*

The results are Mean±S.E.M (n=8); * – Indicates a significant difference from Group II, P<0.05.

**Table 7:** Effect of bitter honey on HDL and LDL Levels.

Treatment group	HDL on day 0 (mmol/L)	HDL on day 28 (mmol/L)	LDL on day 0 (mmol/L)	LDL on day 28 (mmol/L)
**Group I**	0.97±0.02	1.00±0.01	0.70±0.03	0.67±0.03
**Group II**	0.86±0.01	0.72±0.02	1.14±0.04	1.30±0.04
**Group III**	0.84±0.02	0.96±0.01*	1.17±0.03	0.78±0.05*
**Group IV**	0.85±0.03	0.98±0.02*	1.18±0.05	0.85±0.05*
**Group V**	0.81±0.02	0.99±0.01*	1.20±0.03	0.82±0.07*

The results are Mean±S.E.M (n=8); * – Indicates a significant difference from Group II, P<0.05.

Similarly, the treatment of diabetic rats with conventional medication and bitter honey resulted in a decrease in VLDL levels when compared to untreated diabetic rats. The total cholesterol levels of diabetic rats treated with bitter honey in both doses (200 and 400 mg/kg b.w.) and glibenclamide-treated diabetic rats reduced significantly (Table 8). Diabetic untreated rats showed elevated SGOT and SGPT enzyme levels compared to normal rats (Group I). Bitter honey (200 mg/kg b.w. and 400 mg/kg b.w.) and standard drug-treated rats had significantly (P<0.05) lower SGOT levels on day 28 (Table 9). The SGPT levels of bitter honey-treated Group V rats at 400 mg/kg dose b.w. reduced significantly (P<0.05), while no improvement in the SGPT levels of diabetic rats was reported in Group III and Group IV diabetic rats treated with standard glibenclamide and 200 mg/kg b.w. bitter honey, respectively (Table 9). The levels of creatinine and urea were increased in Group II untreated diabetic rats (Table 9). It was observed that oral administration of bitter honey in group IV and group V diabetic rats significantly decreased (P<0.05) the levels of urea after 28 days of treatment.

**Table 8:** Effect of bitter honey on VLDL and total cholesterol levels.

Treatment group	VLDL on day 0 (mmol/L)	VLDL on day28 (mmol/L)	Total cholesterol on day 0 (mmol/L)	Total cholesterol on day 28 (mmol/L)
**Group I**	0.13±0.004	0.12±0.006	0.70±0.03	0.67±0.03
**Group II**	0.18±0.008	0.22±0.001	1.14±0.04	1.30±0.04
**Group III**	0.18±0.006	0.16±0.006*	1.17±0.03	0.78±0.05*
**Group IV**	0.20±0.004	0.16±0.004*	1.18±0.05	0.84±0.05*
**Group V**	0.18±0.006	0.15±0.056*	1.20±0.03	0.82±0.06*

The results are Mean±S.E.M (n=8); * – Indicates a significant difference from Group II, P<0.05.

**Table 9:** Effect of bitter honey on serum liver and kidney biomarkers.

Treatment group	Serum liver and kidney biomarkers on day 28			
	SGOT (U/L)	SGPT (U/L)	Urea (mmol/L)	Creatinine (µmol/L)
**Group I**	184.7±4.57	60.20±3.47	9.27±0.32	0.56±0.03
**Group II**	211.4±4.50	68.40±4.45	12.22±0.36	0.80±0.02
**Group III**	182.0±5.10*	56.20±3.62	10.93±0.30	0.71±0.01*
**Group IV**	164.5±5.37*	63.10±3.20	10.37±0.46*	0.72±0.02*
**Group V**	166.2±4.73*	49.30±4.47*	8.80±0.56*	0.69±0.01*

The results are Mean±S.E.M (n=8); * – Indicates a significant difference from Group II, P<0.05.

**Figure 5:**
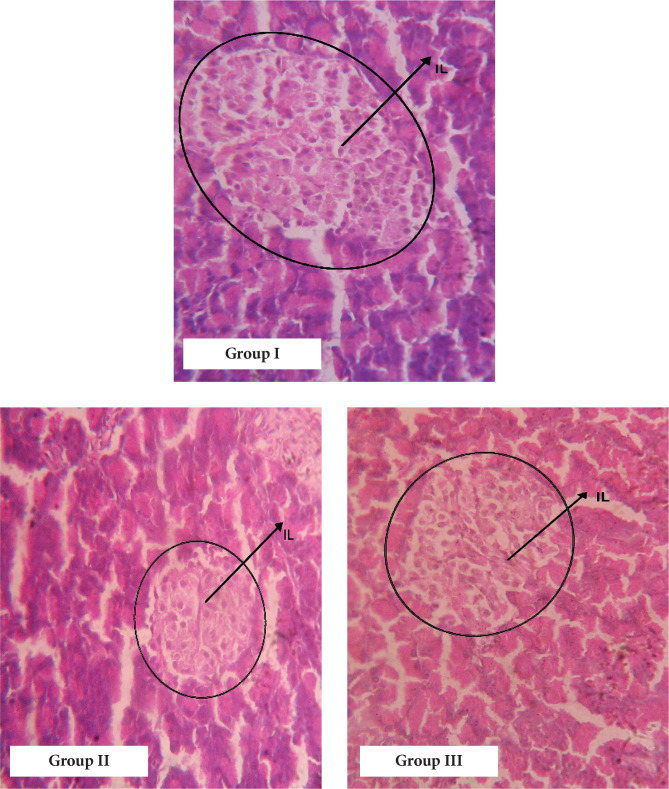

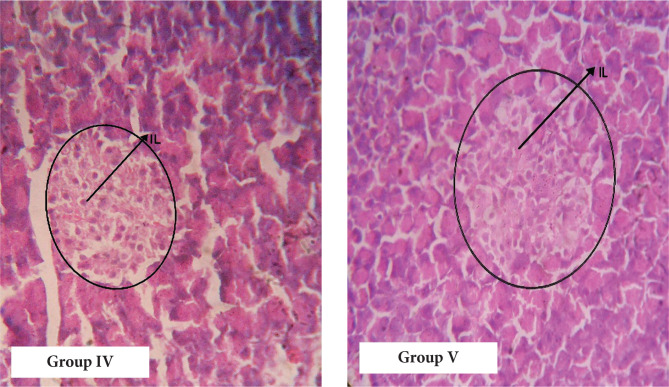
Transverse section (T.S) of the pancreas (400X) representing the histological changes in islets of Langerhans (IL): Group I: Normal histological view of the pancreas; Group II: Shrunken islets and disorganization of pancreatic tissue; Group III: Distorted islet with degenerative change; Group IV: Cells are shrunken with degenerative changes; Group V: Islets appear near to normal.

On the contrary, no significant reduction was observed in urea levels of standard drug-treated diabetic rats. Treatment with bitter honey and standard drug reduced the creatinine levels of diabetic rats significantly (P<0.05). Histopathological examination of the size of pancreatic islets in group II rats was small compared to non-diabetic rats (Figure 5). The islet size in bitter honey-treated groups was improved compared to diabetic untreated rats.

## DISCUSSION

The quality of bitter honey used in the current study was previously evaluated by the authors using standards like FSSAI [30]. The presence of zinc and copper in bitter honey indicated its nutritional importance, and the absence of heavy metals like lead, cadmium, and nickel indicated its safety for consumption. The zinc and copper content of bitter honey was similar to that reported in honey obtained from the canary island in the range of 1.18–1.89 mg/kg and 0.20–0.44 mg/kg, respectively [20]. The drugs which exert an inhibitory action against the enzymes of carbohydrate metabolism are considered effective in managing diabetes [31]. Bitter honey was found to be a potent inhibitor of *alpha-amylase* and *alpha-glucosidase* enzymes. Acarbose exhibited 42.69% *alpha-amylase* inhibition at a concentration of 0.02 mg/mL, while bitter honey at a higher concentration of 0.2 mg/mL exhibited a percentage inhibition of 38.45%. Similar studies reported a 44.34% *alpha-amylase* inhibition of raw *Apis dorsta* honey at 500µg/mL with an IC50 value of 870.5 µg/mL [23]. In the current study, the *alpha-glucosidase* inhibition of bitter honey showed 16.46% inhibition against *alpha-glucosidase* enzyme at a concentration of 0.1 mg/mL while standard exhibited 112.86% inhibition at the same concentration indicating that the potency of bitter honey was less compared to the standard. Stingless bee honey obtained from coconut reported 68.32% of *alpha-glucosidase* inhibition at 100 µg/mL and an IC50 value of 77.6 µg/mL. Honey obtained from star fruit reported an IC50 value of 100 µg/mL.

The safe doses of drugs determined after performing animal acute toxicity studies can be translated to humans [32]. In the current study, the nontoxic nature of bitter honey was assured by performing acute toxicity studies. Streptozotocin- Nicotinamide is a well-established model used to induce type 2 diabetes in experimental animals. This is characterized by the coupling of the deficiency with insulin resistance. STZ causes cytotoxicity of pancreatic beta cells by nitric oxide release, thereby reducing the concentration of islet pyridine molecule. STZ given after Nicotinamide limits pancreatic damage by causing partial deficiency of insulin.

Diabetes induced by STZ is usually accompanied by a severe decrease in body weight, hyperglycemia, hyperphagia, polyuria, and polydipsia [33]. The basic mechanism involved in hyperglycemia involves an overproduction of glucose and its decreased utilization by the tissues. An increased FBG level and loss of body weight, and signs of polyuria and polydipsia in the current study indicated that diabetes was effectively induced in rats. The decrease in body weight is due to the degradation of connective tissue caused by the administration of STZ [33]. In diabetic rats, bitter honey treatment did not produce a beneficial effect on body weight. Honey promotes lipolysis, prevents lipogenesis, and improves the metabolic rate that contributes to body weight loss, while sulfonyl urea increases body weight by decreasing the excretion of glucose in urine [34, 35]. The conventional antidiabetic drug Glibenclamide is commonly used for comparing the potency of antidiabetic compounds in the STZ-induced antidiabetic study [36].

In diabetic rats, bitter honey treatment showed a dose-related decrease in FBG levels. A similar reduction in the FBS levels was previously reported in tulang honey-fed streptozotocin-induced diabetic rats for 28 days [37]. Honey administered orally in Sprague-Dawley rats for about 52 weeks elevated HDL levels [38]. A randomized and controlled clinical study of natural honey consumption for 8 weeks reported a beneficial effect on reducing FBG levels in diabetic individuals [39]. The antidiabetic effect produced by bitter honey can be due to the presence of fructose, one of the main constituents of honey. Fructose stimulates glucokinase, which helps in the hepatic uptake of glucose and glycogen storage [40]. In diabetic rats, the presence of zinc and copper in honey was reported to lower the levels of lipid peroxidation and blood glucose [17, 18]. The result of the present study indicated the presence of zinc and copper in bitter honey.

Decreased HDL and accumulation of plasma LDL, TC, VLDL, and TG levels are commonly associated with diabetes mellitus. The major risk factors of cardiovascular disease are elevated LDL, TC, and VLDL levels. Increased HDL reduces cardiovascular complications by facilitating the peripheral liver transport of cholesterol [41, 42]. Diabetic rats fed with both dosages of bitter honey had a substantial decrease (P<0.05) in TG, TC, VLDL, and LDL levels and an increase in HDL. The mechanism by which bitter honey exerts a hypolipidemic effect is unknown but possibly due to the presence of plant-derived phytochemicals. An increased HDL level and decreased FBG levels, total cholesterol, and LDL levels were reported after consuming natural honey for 30 days in human subjects [43].

The elevated levels of SGOT, SGPT, urea, and creatinine are liver and kidney dysfunction markers induced by the administration of STZ-NA. Bitter honey-treated diabetic rats in group V showed a decrease in SGOT and SGPT levels, whereas diabetic rats treated with a dose of 200 mg/kg b.w. of bitter honey did not produce any reduction in the SGPT levels. SGOT levels were reduced in bitter honey-treated rats of Group IV and Group V compared to diabetic rats without any treatment. Bitter honey may exert a hepatoprotective effect in a dose-dependent manner. Bitter honey also reduced the urea and creatinine levels of diabetic rats indicating its nephroprotective effect. A reduction in SGOT, SGPT, urea, and creatinine was also reported after administering bitter gourd honey in diabetic rats. Similar results were reported in carob honey and bee honey in rat liver and kidneys [44–46].

The isolation of phytochemicals present in bitter honey responsible for the antidiabetic activity was not carried out. Hence future research should isolate the phytochemical compounds in bitter honey responsible for antidiabetic activity using various analytical methods.

## CONCLUSION

The current study revealed the antidiabetic activity of bitter honey in Streptozotocin- Nicotinamide-induced diabetic rats. Bitter honey treatment also improved the lipid profile levels in diabetic rats, which indicated its protective effect in diabetic-associated dyslipidemia. The presence of phytochemicals responsible for antidiabetic activity was reported in our previous studies. Hence, it can be concluded that bitter honey can be used as an alternative in managing diabetes and its related complications. Moreover, extensive pharmacological studies are required to identify the exact mechanism responsible for antidiabetic activity.
